# A Study on the Gaseous Benzene Removal Based on Adsorption onto the Cost-Effective and Environmentally Friendly Adsorbent

**DOI:** 10.3390/molecules28083453

**Published:** 2023-04-13

**Authors:** Kaan Isinkaralar

**Affiliations:** Department of Environmental Engineering, Faculty of Engineering and Architecture, Kastamonu University, Kastamonu 37150, Türkiye; kisinkaralar@kastamonu.edu.tr

**Keywords:** air pollutants, gas treatment, environmental protection, reversible adsorption, sustainable porous carbons

## Abstract

Removal of benzene is essential for human and environmental health because it has toxic and hazardous properties at various concentrations. Theseneed to be effectively eliminated with carbon-based adsorbents. PASACs, carbon-based adsorbents obtained from using the needles of *Pseudotsuga menziesii*, were produced by optimized HCl- and H_2_SO_4_-impregnated approaches. Regarding physicochemical structure, the optimized PASAC23 and PASAC35 with surface areas of 657 and 581 m^2^/g and total pore volumes of 0.36 and 0.32 cm^3^/g showed ideal temperatures of 800 °C. In order to investigate and compare internal benzene removal efficiency, PASAC23 and PASAC35 were studied separately. Initial concentrations were found to range from 5 to 500 mg/m^3^, and between 25 and 45 °C. The removal rate of benzene by PASAC23 and PASAC35 was 97 and 94% at low concentrations, respectively. While the highest capture amount for PASAC23 and PASAC35 was found to be at 25 °C with 141 and 116 mg/g, the adsorption capacity decreased to 102 and 90 mg/g at 45 °C. The holding capacity decreased between 22.41 and 27.66% due to increasing temperatures. After five cycles of PASAC23 and PASAC35 regeneration, we found that they could remove 62.37 and 58.46% of benzene, respectively. These results confirmed that PASAC23 is a promising environmentally adsorbent for effectively removing benzene with a competitive yield.

## 1. Introduction

The present world has been exposed to gaseous benzene, although the degree to which this has occurred varies depending on the level of air pollution exposure through inhalation [1]. The pollution of megacities can be explained by rising emissions of industry and traffic combined with irregular and uncontrolled urban growth. In these environments, air pollutants manifest themselves at a detectable level [2,3]. While benzene is common and diverse, it is also readily available in high concentrations. Depending on the environment and conditions it is found in, benzene has a direct negative impact on human health since it is spread and transported. According to World Health Organization (WHO) reports, benzene is a halogenated aromatic compound that can be found widely and which has been associated with some acute and long-term adverse health effects, such as cancer and aplastic anemia [4,5]. A variety of diseases, cancers, and death may occur in humans due to current ambient air toxic levels in Group I, as determined by the WHO [6,7]. Initially, benzene can be emitted from dominant sources, including motor vehicles [8], industries [9], residences [10], dry-cleanings [11], indoor dust [12], cigarette [13], chemicals [14], natural and anthropogenic [15]. Benzene emissions from household items such as decorative, wood, and veneer composite objects are associated with contact time and concentration, depending on their concentrations [16].

Various studies have shown that adsorption can effectively remove a comprehensive variety of benzene in the gas phase [17,18]. However, various adsorbents and technologies are available to treat gaseous benzene. Their performance has been investigated for its low energy consumption, ease of operation, and high removal efficiency [19,20,21]. Today, varieties of activated carbons with different physicochemical properties are selected according to benzene concentration and amount, as well as its duration in the indoor air. The use of activated carbons comes to the fore since ventilation, the most effective method for removing benzene gas indoors, is not always possible, and outdoor air levels may be worse than those of ambient air [22]. To deal with this, for example, activated carbons with a surface area of 600–900 m^2^/g can be used in the home or office. A surface area of 1000–3500 m^2^/g can be used for industrial applications [23,24,25]. Some types of activated carbons can be modified to capture the particular pollutant according to the intended use [26]. Activated carbons are preferred because of their many features, such as popularizing the benefits, which is the basis of the production principle of air-cleaning devices, being able to be used without changing for a long time, and being cheap and easy accessibility [27]. Due to all these ergonomic and practical capture features, the demand for activated carbon is increasing worldwide. The production of green and environmentally friendly adsorbents, which are cheaper and have higher performance than other techniques, is being researched [28,29,30]. The recovery and usability of benzene captured on activated carbon can also be gained by desorption. This has paved the way for the reusability of charged benzene gas in industries producing high concentrations of it as a product. However, for the conditions to be optimum and for the desorption conditions to be realized, the physicochemical conditions of the adsorbent must be suitable.

The most abundant non-native tree species, *Pseudotsuga menziesii*, is also supposed to be more resistant to wind throw. Still, solid storms or wild animals can damage multiple organs and needles [31]. However, storm susceptibility could be higher in the case of more waste being generated from trees in the form of debris complex from Douglas fir forests. Since the amount of waste generated at the regional scale is relatively high, ways to recycle it in an environmentally friendly manner must be sought. Large-scale destruction is used in forestry and woodworking, but no precise method exists with which to reuse small-scale waste. As such, investigating its suitability for making activated carbon has led to quite a reasonable understanding of the environment being developed. Considering the producing activated carbon which it protects nature for, especially in global quantities.

The purpose of this paper is to evaluate dried needles and the easy production of a carbon-based adsorbent using lignocellulosic biomass. First, as a novel forestry waste, biomass conversion was a low-cost and environmentally friendly production of a high-value biomaterial using *P. menziesii*. PASAC was prepared using hydrochloric acid and with sulfuric acid activation as a reinforcing agent. Various characterizations determined the physicochemical characteristics of PASACs. Subsequently, they were incorporated into the batch reactor in order to remove benzene, with a high performance attained from the gas environment. With the model gas benzene molecules, it should be noted that physiochemical factors optimized the notations PASAC23 and PASAC35 adsorbent in order to vouchsafe irresistible advantages such as re-generatability, recyclability, and high adsorption capacities.

## 2. Results

### 2.1. Characterization Results

The effects of the activation conditions which were involved in the production of PASACs on their physicochemical structure varied. Differences occurred due to the differences in the construction of 50 adsorbents between PASAC1 and PASAC50, their interactions, and variations of the groups on their surfaces. As a result of the activation with HCl, 1.58 times more micropore volume was obtained compared to activation with H_2_SO_4_, while an increase of 0.28 times was achieved in the total surface area. While the carbon content of the raw samples was 42.56%, the highest carbon content found after activation with HCl was 70.83 and 67.94% in PASAC23 and PASAC35, respectively. There was a significant decrease in oxygen content, from 50.25 to 19.64%, after activation. However, the increase in nitrogen content after activation ranged from 2.56 to 9.45%. The main reason for this was the uninterrupted inclusion of N_2_ gas into the system so that the environment was inert during activation. As a result of the increase in carbon in the pores, the variations in elemental values were realized, as shown in Table 1.

An awareness has emerged as to the contents of moisture, volatile substant, fixed carbon, and ash given in Table 2, which were determined according to the PASB method. In particular, volatile substances decreased from 73.27 to 17.27 and 21.61%. The volatile substances in the raw sample moved away from the main structure formed in the carbon-based adsorbent, and fixed carbon replaced them. Although the fixed carbon amount was 15.42% in PASB, it increased to 74.38% and 68.24% in PASAC23 and PASAC35, respectively.

Table 3 shows the size of the spatial and volumetric growths in the pore structure before and after the activation process. The S_BET_ value of PASB, which was 3 m^2^/g before activation, increased to 657 and 581 m^2^/g in PASAC23 and PASAC35. Despite the lack of pore volumes of PASB, the total pore volumes after activation with HCl and H_2_SO_4_ were 0.36 and 0.32 cm^3^/g. The striking point in the increase here is that, although the total surface area of PASAC23 was large, the micropore volume did not differ significantly from PASAC35. The efficiencies of adsorbents with such close micropore volumes in benzene removal were also compared so that the total surface area or the micropore volume affected the efficiency.

**Figure 1:**
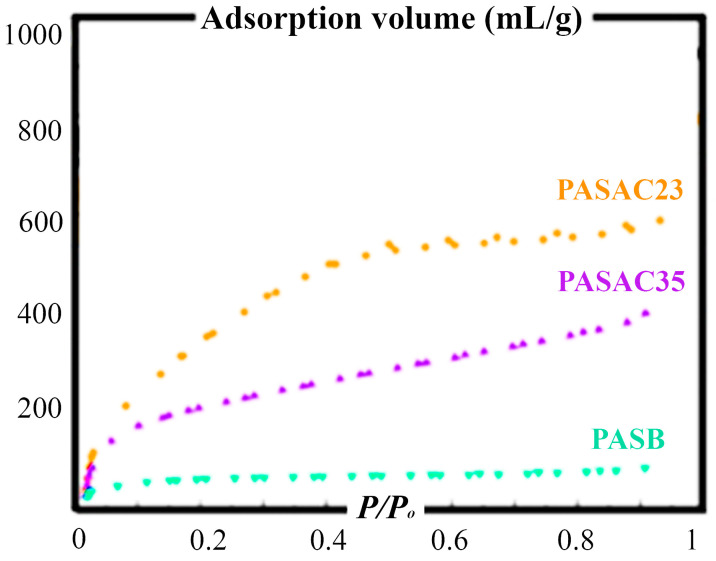
Adsorption/desorption isotherms of N_2_ at 77 K of PASB, PASAC23 and PASAC35.

Figure 1 shows adsorption–desorption isotherms of N_2_ at 77 K for PASB, PASAC23 and PASAC35 and can be used to obtain the shape and behavior of the N_2_ adsorption isotherms. PASACs were degassed at 300 °C for 3 h. All the isotherms belonged to microporous material features whereby a type I isotherm at low relative pressures (*P*/*P*_0_) and type IV isotherm at intermediate and high relative pressures presented a hysteresis loop for the directive of the International Union of Pure and Applied Chemistry (IUPAC). The main point distinguishing type IV and type I was the capillary condensation which occurred at relative pressure range *P*/*P*_0_ > 0.4 in the mesopore region due to the hysteresis loop of type IV being formed by isotherms.

**Figure 2:**
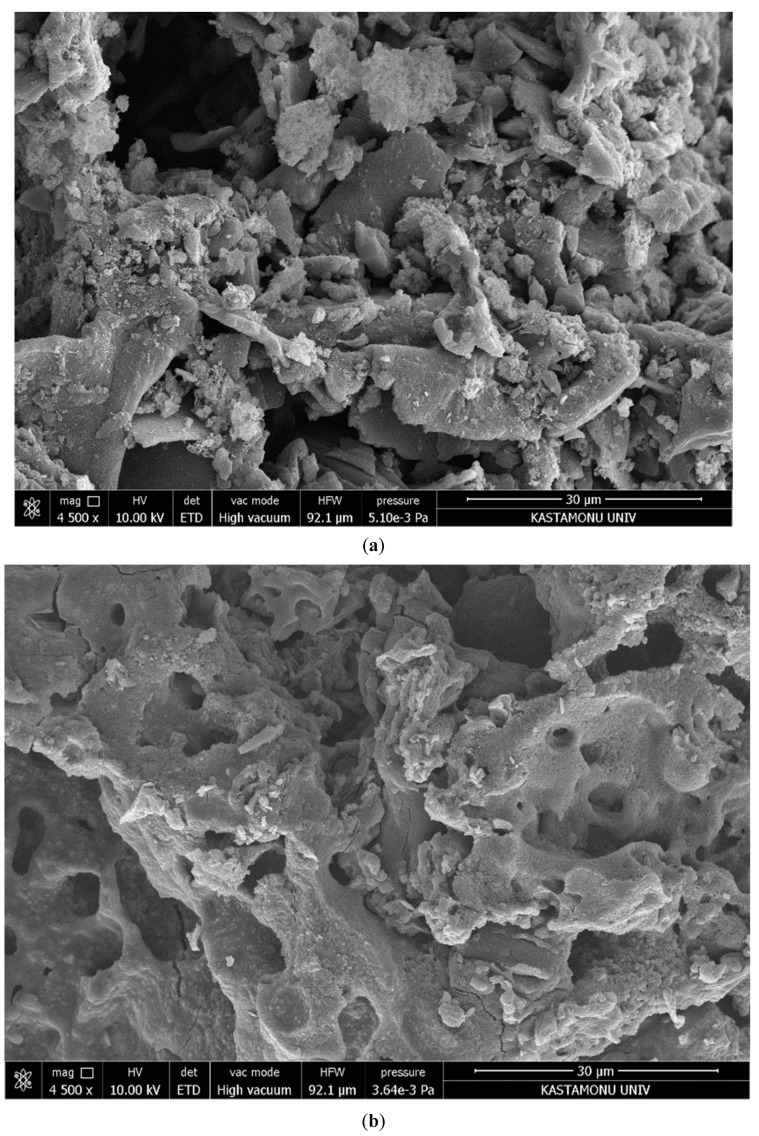

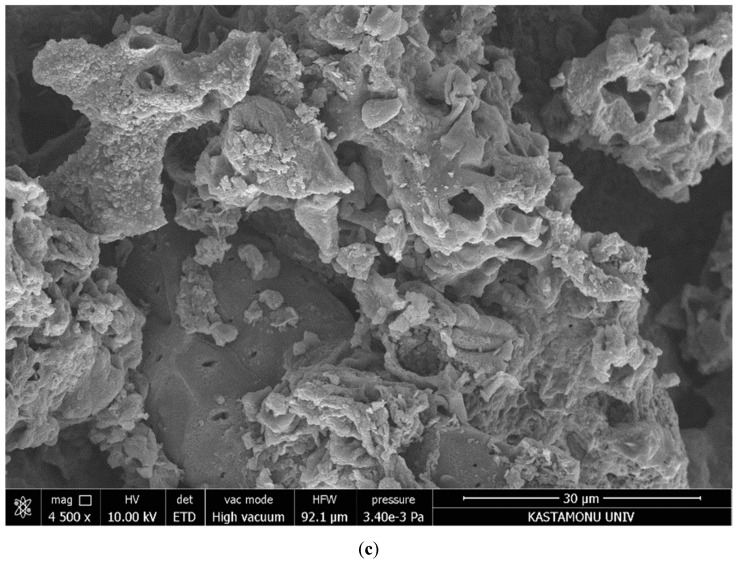
Representation of SEM images of (**a**): PASB, (**b**): PASAC23 and (**c**): PASAC35.

Figure 2 corresponds to the SEM images in that Figure 2a has no porosity, but Figure 2b,c have porosity via cracks, crevices, and some grains in various sizes after activation. It is understood that the inhomogeneous, scattered, and independent structures formed were observed at the optimum operating condition, with 4500× magnification. The formation had a shape which was suitable for the lignocellulosic contents. Compared to adsorbents, PASAC23 was found to have more pores and a looser texture, although both reflected biomass material’s lignocellulose properties [32,33]. A previously reported study by Isinkaralar & Turkyilmaz [34] using *R. communis* as lignocellulosic material reported a similar porous and heterogenous surface for PASACs.

**Figure 3:**
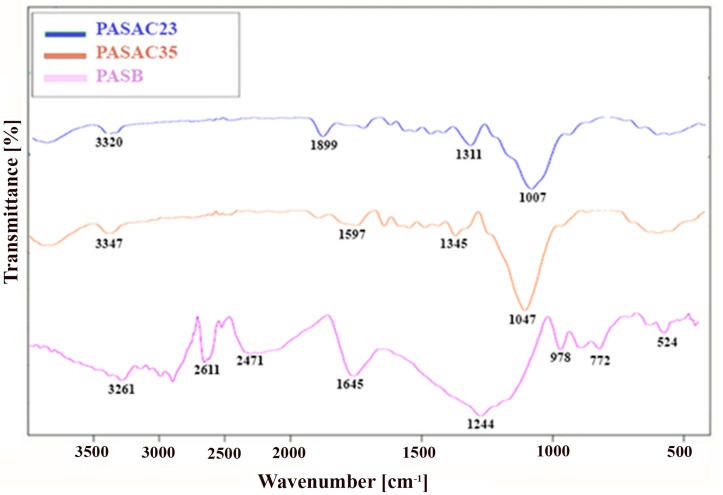
FT-IR spectra of PASB, PASAC23 and PASAC35.

To reveal the FT-IR spectra of PASB, PASAC23, and PASAC35 are indicated in Figure 3. The characteristic FTIR peaks show that the samples peaked for whole functional groups such as carboxylic, hydroxyl, and carbonyl groups due to the instability of acidic groups at high temperatures and the large amounts of cellulose and hemicelluloses. The broad band at around 3347 and 3261 cm^−1^ was attributed to the O–H stretching of alcohol and phenol groups [35]. The sharp peaks at 2611–2471 cm^−1^ were associated with the C–H group stretching and O–H deformation vibrations in alkane groups [36]. The N–H in-plane bending and C–N stretching vibration peaks at 1899–1597 cm^−1^ was likely related to amine groups. The C–O–C stretching vibration at 1047 cm^−1^ occurred due to alcohols, phenols, or ethers [37]. The characteristic FTIR absorption bands of cellulose, less than 1007 cm^−1^, also appeared in the spectra of PASB due to the characteristic of lignin structures. Several researchers also reported similar effects on the functional groups in lignin-based adsorbents [38,39,40].

**Figure 4:**
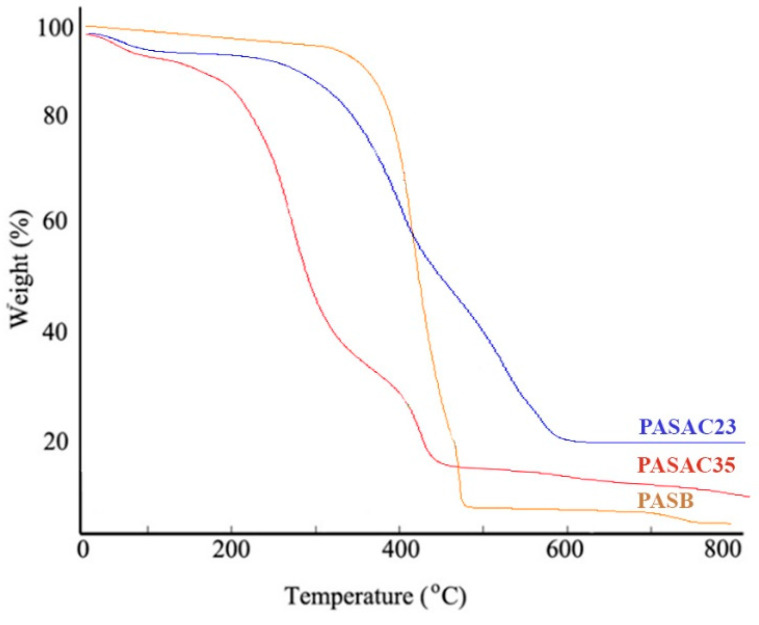
TGA curves of PASB, PASAC23 and PASAC35.

Figure 4 illustrates the TGA curves, which were determined to examine the thermal stability of PASB, PASAC23, and PASAC35 under the processing temperature prior to attaining their dryness at 80 °C for 3 h. The three significant stages of weight loss were as follows: (i) there was a loss of water at 100 °C and at the first stage at 228 °C, which indicates the decomposition of the oxygen-containing functional groups on the surface; (ii) during the second stage, we observed the decomposition of hydroxyl, epoxy and carboxylic groups, with a loss of oxygen-containing groups at temperatures between 280 and 420 °C [41]; (iii) carbon backbone decomposition occurred after at 430 °C [42]. The PASB consisted of cellulose, hemi-cellulose and lignin. Cellulose decomposed in the temperature ranges of 486–615 °C.

### 2.2. Benzene Adsorption Capacities of the PASACs

We attempted to determine the removal efficiency of the PASAC23 and PASAC35 adsorbents used in the study at 25, 35 and 45 °C and the number of benzene molecules attached to adsorbents. The behaviors of benzene molecules were observed at different temperatures and residence times.

### 2.3. Effect of Initial Concentration

**Figure 5:**
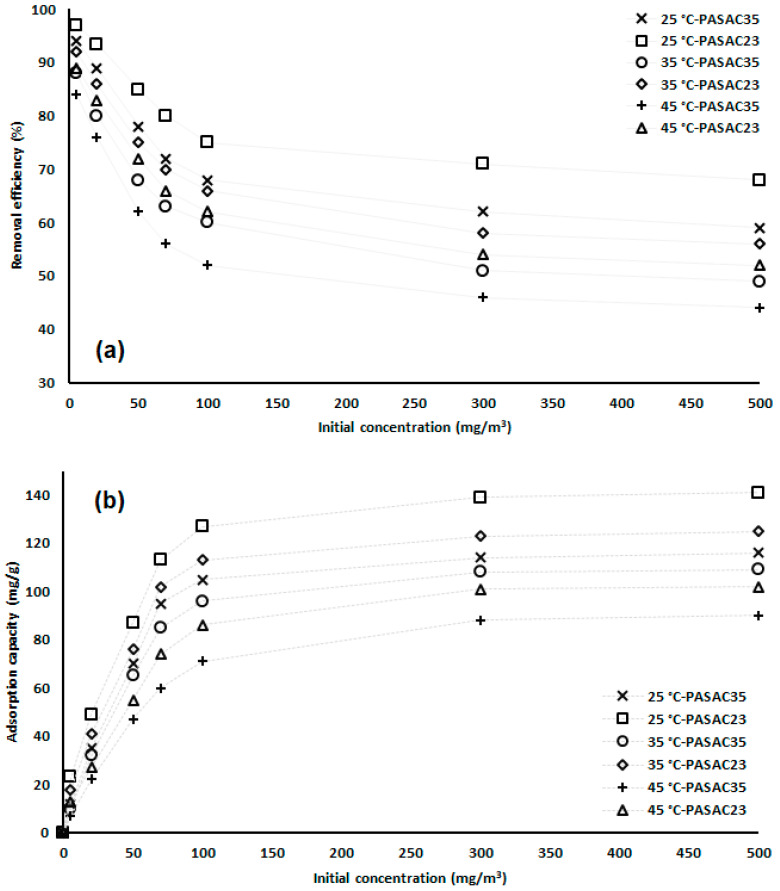
Initial concentration and its effect on adsorption (**a**): removal efficiency and (**b**): adsorption capacity.

The adsorption efficiencies obtained against the initial concentration, given in Figure 5a, were obtained for PASAC23 and PASAC35. The increase in the concentration amount caused a severe decrease in the yield, from 25 to 45 °C. The main reason for this was that benzene gas, whose solubility increased at room temperature, first increased and then decreased as the temperature increased. The highest yield was obtained at 5 mg/m^3^, while the lowest yield was obtained at 500 mg/m^3^. The decrease in efficiency was gradually seen in three stages in total; 1st stage decrease was 5–50 mg/m^3^, 2nd stage decrease was 50–100 mg/m^3^ and the last decrease was significant decreases after 100 mg/m^3^. The highest values for PASAC23 and PASAC35 were 97 and 94%, while the lowest values were 52 and 44%. The increase in temperature decreased the yield of PASAC23 by 46.39%, while the decrease in PASAC35 was 53.19%.

The maximum adsorption capacity shown in Figure 5b depended on increasing benzene concentration. Still, considering the amount of PASAC23 and PASAC35 used, the pores were filled, and there was no room for more benzene molecules. Adsorption capacity is generally divided into two groups; the first one is an adsorption capacity of 0–50 mg/m^3^, and the highest adhesion capacities were obtained here; the second group is at 50–500 mg/m^3^, which is a holding capacity, the adhesion rate and amount remained relatively low here. The main reason for this was the rapid filling of the pores and the inability of their surface areas to respond to benzene molecules. While the highest capture amount for PASAC23 and PASAC35 was found at 25 °C with 141 and 116 mg/g, the adsorption capacity decreased to 102 and 90 mg/g at increasing temperatures up to 45 °C. There was a loss of between 22.41 and 27.66% holding.

### 2.4. Effect of Contact Time

**Figure 6:**
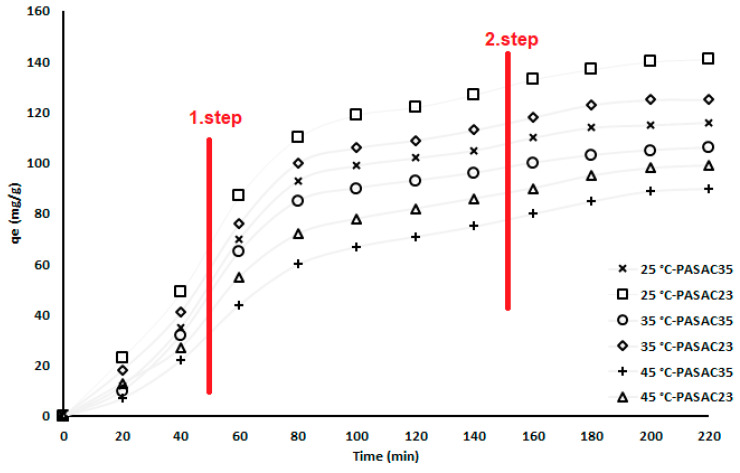
Influence of contact time on the performance of benzene adsorption.

The curve shown in Figure 6 gives the qe (mg/g) amounts of PASAC23 and PASAC35 that change over time. The time-dependent deceleration of the curve slope indicates that the pores of PASAC23 and PASAC35 were filled in level as 1. step and 2. step, and that the benzene molecule reduced the amount of capture. Dividing the increase in the amount of qe into two steps, the results indicated that the speed was at a maximum in the first step and that the rate started to decrease and ultimately stabilized in the second.

### 2.5. Regeneration Process

**Figure 7:**
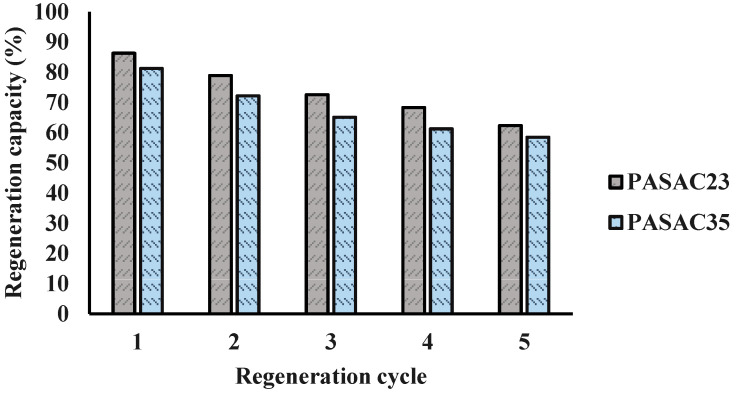
Regeneration capacity of PSAC23 and PSAC35.

Considering the environmental impact and economic values of recyclability, the spent PASAC23 and PASAC35 reusability test was carried out. Its results are outlined in Figure 7. The absorbents PASAC23 and PASAC35 retained 62.37 and 58.46% of their adsorption capacity after five adsorption–desorption cycles. The results show they can be reused continuously without significant loss, 28% with lost after the first cycle. It is known that the attachment of benzene molecules to the micropores of PASACs is physical rather than chemical. Therefore, in this process, a small part of the adhesion in the adsorption event was released into the environment through PASACs over time and destroyed by benzene molecules.

## 3. Discussion

Exposure to benzene may have a notable effect on human health due to its adverse impacts, with carcinogenic effects and the potential to cause hematological disorders [43,44]. Sources for benzene in the ambient environment may include the infiltration of indoor spaces and benzene emitting utilized in the production of many materials [45]. Due to ubiquitous chemicals, several daily emission activities increase the number of benzene molecules taken into the body from inhalation [46]. Thus we need to reduce benzene removal applications [47]. Benzene management can be accomplished by adsorption, which is one of the most successful techniques, with high flexibility. Activated carbon is the most familiar adsorbent, but its usage is limited because it has a high cost and faces adversities in relation to disposal and regeneration [48]. For this reason, agricultural, forestry-based, and lignin-containing wastes are highly promising adsorbents due to their easy-to-handle, large, porous, loose structure. They have been as a carbon-based adsorbents by several studies for the removal of volatile organic compounds, including benzene [49,50,51,52]. Liang and Chen [53] investigated benzene adsorption behaviors onto AC, OAC-5 and AC/FeS_2_. They reported that the AC/SPS/FeS_2_ way offered a thriving procedure for devastating benzene that had been sorbed onto AC or desorbed into the liquid form in the case of the AC/SPS system. Khan et al. [54] reported that the use of several biochars, derived from KOH activation, was tried to use the removal of 50 ppm gaseous benzene. The highest maximum adsorption capacities were 144 mg/g for MGB, 29.3 mg/g for GBS, 10.8 mg/g for CBN, and 79.1 mg/g for ACd212. Wang et al. [55] designed a PAC-based denuder system for benzene vapor adsorption at 100, 200, 300, and 500 mL/min. The BET surface area of PAC sorbents and its capacity were obtained at 1071 and 929 m^2^/g; they were found to vary from 20 ± 1 to 43 ± 2 and 0.32 ± 002 to 0.86 ± 0.02 in packed and denuder systems. Similarly, the conclusion that benzene adsorption onto activated carbon is a widespread application was proposed independently by Li et al. [56] and Ha et al. [57]. Shen et al. [58] developed BC*x*-*y* using banana peel by KOH for benzene and toluene vapor adsorption. The performances of benzene and toluene with BC3.5-800 were 27.55 mmol g^−1^ and 23.82 mmol g^−1^. A more detailed investigation is warranted to understand the benzene adsorption processes better and to describe benzene’s molecular diffusion mechanism through the interpores of porous PASAC23 and PASAC35.

## 4. Materials and Methods

### 4.1. Materials and PASAC Yield

Needles of *Pseudotsuga menziesii* were provided by a local forest in Kastamonu, Türkiye. They are found abundantly in winter months as forest waste due to adverse weather conditions. They were collected from nature and brought to the laboratory. Benzene (99.8% C_6_H_6_), hydrogen chloride (37% HCl), and sulfuric acid (98% H_2_SO_4_) were of high purity and were supplied by the Merck company, Germany. Benzene analytical standard and Tenax^®^ TA stainless steel sorbent tubes (35/60 mesh) were purchased from Supelco^®^. Additionally, deionized water was utilized for the preparation of the solution, synthesis, and purification. Equation (1), used to calculate the efficiency in the production of PASACs, shows the inputs and outputs by weight.

(1) $$\mathrm{PASAC}\;\mathrm{yield}\;\left(\%\right):\;\frac{Weight\;of\;PASAC}{Weight\;of\;PSB}\times 100$$

### 4.2. Environmentally Adsorbent Preparation

To retrieve the moisture inside the needles, they were dried in an oven 3 times in total, respectively. Then, (i) they were left in the oven for 3 weeks at 60 °C to dry before starting the process. In order to reduce the size of the dried needles, they were pass-through reduced to 2–3 cm intervals. (ii) In the second drying process of the reduced needles, they were left to dry in an oven at 60 °C for one week. The samples taken from the oven were powdered by placing them in the ring grinder; (iii) the powdered samples were subjected to an oven at 60 °C. After the drying processes, a total of 50 polyethylene samples were weighed and put into containers at 20 g portions. HCl and H_2_SO_4_ solutions started the chemical activation process. They were used to enhance the adsorption performance of the PASB. The PASB was soaked in HCl and H_2_SO_4_ solutions in 1:1, 1:5, 1:7, and 1:10 wt%. The prepared mixtures were sequentially impregnated. For the impregnation process, (i) 1:1 wt% HCl was poured onto a 20 g raw sample and mixed thoroughly for 30 min. (ii) The solution was stirred at 100 rpm continuously for 30 min in an automatic heater set at 150 °C. (iii) This was kept at room temperature for 24 h to achieve a constant weight. The same processes were carried out for HCl and H_2_SO_4_ in other ratios, and a total of 50 samples ready for carbonization were obtained.

The operating steps of the reactor, which was produced from high-temperature-resistant stainless steel material used in the carbonization process, were, respectively (i): the high purity N_2_ inlet of the temperature-adjusted reactor was connected first, ensuring that the flow was uninterrupted in an empty way 100 mL/min; (ii) PASACs were put into the reactor, a process which first took place at 500 °C in a temperature-controlled chamber. The process continued for 120 min in N_2_ flow. (iii) After 120 min, the products were left to cool for 2 h under continuous N_2_ flow. The same processes were applied up to PASAC50 and the reactor’s temperature was increased at a heating rate of 10 °C/min until it reached 600, 700, 800 and 900 °C, after which it was held for 2 h. To eliminate the remaining impurities and unreacted HCl and H_2_SO_4_, the PASACs were washed with 0.1 M NaOH solution and neutralized with a copious amount of hot deionized water. The optimized PASAC23 and PASAC35 had surface areas of 657 and 581 m^2^/g, and total pore volumes of 0.36 and 0.32 cm^3^/g and thus showed an ideal temperature of 800 °C. The proximate and ultimate analyses were performed using the American Society for Testing and Materials (ASTM) standard test procedures of E872-85 [59], E871-82 [60], and E1755-01 [61], respectively.

### 4.3. Structural Characterization

After the main structure had been, it was scrutinized employing a Fourier transform infrared (FT-IR) spectrometer (PerkinElmer, Waltham, MA, USA) in the range of 380–4000 cm^−1^. This was performed by incorporating the sample with the KBr disk (5% of the mixture). In order to characterize the composition, the prepared PASACs were coated with gold for analyses with scanning electron microscope (SEM, FEI Quanta FEG 250, Oregon, USA). Nitrogen adsorption, as assessed with the Brunauer–Emmett–Teller (BET) method, was used as a measure for the determination of the resulting surface area using Quantachrome (Nova, 2000e Quantachrome Instruments, USA). Additionally, the pore diameter was obtained by using the Barrett–Joyner–Halenda (BJH) method, and carbon (C), hydrogen (H), nitrogen (N), and oxygen were assessed in the samples. The elemental analyzer (Eurovector, EA3000-Single, Pavia, Italy) was used to determine the (O) content. A thermal gravimetric analyzer (TGA temperatures between 50 and 850 °C losses) was used to determine the thermal degradation ranges under continuous N_2_ flow at a heating rate of 15 °C/min.

### 4.4. Batch Equilibrium Details

**Figure 8:**
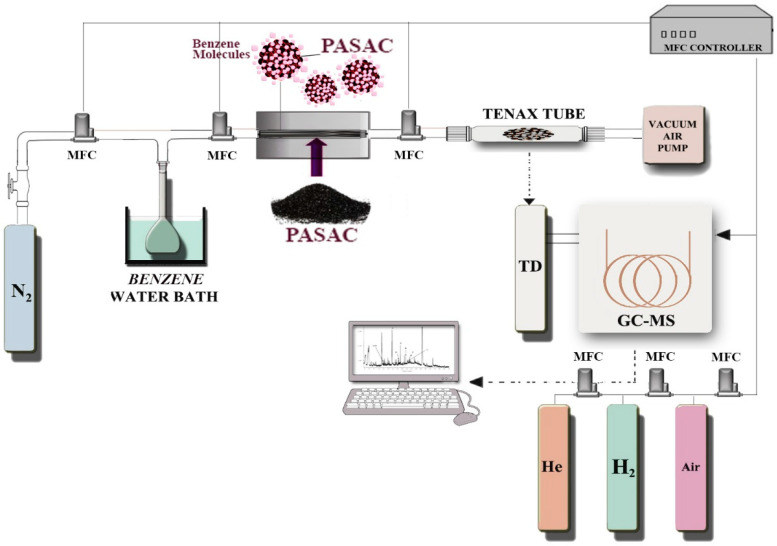
Schematic diagram of benzene removal.

This is the process by which the system is expected to become stable, which is the basis of the working principle of the experimental setup prepared for the removal of benzene gas in Figure 8.

During this process, adjusting the waiting time, the temperature in the system, the adsorbent dose, and the vacuum pump flow rate were crucial, as these were the basic operating parameters by which the system became stable. The system’s stability at the specified basic parameters was ensured before loading various levels of benzene into the batch reactor. Then, (i) we fixed the inside of the system to an initial concentration of 5 mg/m^3^, and an adsorbent weight of 75 mg; (ii) we set the homogeneous distribution of the temperature level of 25 °C in the reactor; (iii) we ensured periodic sampling up to 220 min; and (iv) the continuity of the vacuum air pump was 80 mL/min. It was ensured that benzene gas was collected in Tenax tubes according to USEPA method TO-17 [62]. The four steps were adjusted to 20, 50, 70, 100, 300, and 500 mg/m^3^ at 35 and 45 °C, respectively. Pre-control and cleaning of the used Tenax tubes and concentrations of collected gases were carried out using a TD-GC/MS (Thermal Desorber, Markes Unity, gas chromatography, Thermo Scientific Trace 1300, Mass detector, Thermo Scientific ISQ QD, MA, USA) and capillary column (TG-624; 30.0 m × 0.25 mm × 1.4 µm) under a mass flow controller (MFC). After completion of the sampling, Tenax tubes were stored at −20 °C and analyzed within 48 h. The experiments were continued by selecting only PASAC23 and PASAC35 due to the high surface area and micropore volume of the carbon-based adsorbents used in the study. The saturated adsorption capacity of benzene was calculated by the adsorption curve and the $q_{\left(mg/g\right)}$ was calculated by Equation (2) as follows [63]:

(2) $$q_{\left(mg/g\right)}=\left(\frac{F\;x\;C_{0}\;x\;10^{-9}}{W}\right)\;\left[\left(\frac{C_{i}}{C_{0}}\;x\;t_{s}\right)-\left(\int_{0}^{t_{s}}\frac{C_{i}}{C_{0}}\;d_{t}\right)\right]$$

where W (g) is the weight of PASAC23 and PASAC35; $C_{i}$ (mg/m^3^) is outflow benzene concentration, mg/m^3^; $t_{s}$ is the time of saturation, $q_{\left(mg/g\right)}$ is the saturated benzene adsorption capacity.

### 4.5. Quality Control and Quality Assurance

The benzene adsorption experiment was validated using the limit of detection (LOD), the limit of quantification (LOQ), and the relative standard deviation (RSD). The blank method was used to specify LOD and LOQ values. Herein, these values were estimated using the concentrations of 10 blank samples (B) as in the triplicate test in Equations (3) and (4).

(3) $$LOD:\;Mean{\;}_{B}+\left[3\times SD_{B}\right]$$

(4) $$LOQ:\;Mean{\;}_{B}+\left[10\times SD_{B}\right]$$

Concentrations of blank samples, LOD and LOQ were 0.00–0.02 mg/m^3^, 0.02 and 0.06 mg/m^3^. The results of benzene concentration were more accurate in all experiments.

### 4.6. Recyclability and Reusability of Spent PASAC

Recyclability and regeneration of PASAC23 and PASAC35 were used in the benzene eliminate test using a 0.05 g dosage of each adsorbent. They were investigated using the thermal desorption method at 10 °C/min to 240 °C and treated under N_2_ flow to evacuate the micropores of the adsorbed benzene molecules deeply. The regenerated PASAC23 and PASAC35 were reused for benzene adsorption and subjected to heat treatment for the next regeneration cycle. This process was repeated five times. The PASAC23 and PASAC35 were applied for another benzene adsorption and calculated to subsequent cycle for regeneration efficiency (RE) in Equation (5) as follows [64]:

(5) $$RE\;\left(\%\right):\;\frac{Adsorption\;capacity\;of\;regenerated\;PASAC}{Adsorption\;capacity\;of\;fresh\;PASAC}\times 100$$

## 5. Conclusions

Airborne benzene, found in most of the daily products used by human beings as a substitute for indoor spaces, is a toxic compound that can be quickly released into the environment in indoor air. Controlling benzene, which is often present in the ambient air due to insufficient and deficient ventilation, is essential as the substance threatens human health in high concentrations. Therefore, removing benzene, which has become very important, with carbon-based adsorbents has yielded very successful results. Choosing a convenient adsorbent is a vital factor in benzene removal, and the correct choice depends on the specific pore structure, surface area, and chemical functional groups. In this context, needles belonging to the lignocellulosic-containing *P. menziesii* species, a natural waste used in this context, were transformed into an effective and successful adsorbent on benzene via chemical activation. The interaction of PASAC23 and PASAC35 with benzene has been investigated and the results showed increased adsorptive capacity. Experiments were executed in order to inspect the effects of the high concentration level and different temperatures on benzene adsorption. The benzene adsorption data revealed that the porosity and surface contact area of PASAC23 and PASAC35 decreased by 22.41–27.66% when loaded in the batch system due to increased temperature. However, if the PASAC23 and PASAC35 were compared based on expected performance parameters such as adsorption capacity, the performance of the PASAC35 was reduced by 0.82–1.4-fold relative to the PASAC23. This reduction can be ascribed to textural, morphological, and active external surface sites of PASAC35. Based on these findings, the capture systems present in this study may enable a reference for potential on-site volatile organic compound adsorption–desorption of PASAC23 and PASAC35 applications.

## Figures and Tables

**Table 1:** Elemental components (wt%) of PASB and PASACs.

Element (wt%)	PASB	PASAC23	PASAC35
C	42.56	70.83	67.94
H	4.71	3.67	2.87
O	50.25	22.17	19.64
N	2.56	3.33	9.45

**Table 2:** Proximate analysis of PASB and PASACs.

Proximate (wt%)	PASB	PASAC23	PASAC35
Moisture	10.66	2.58	5.53
Volatile substant	73.27	17.27	21.61
Fixed carbon	15.42	74.38	68.24
Ash	0.65	5.77	4.62

**Table 3:** Pore structure parameters of PASB, PASAC23, and PASAC35.

Materials	PASB	PASAC23	PASAC35
S*_BET_* (m^2^/g)	3	657	581
V*_total_* (cm^3^/g)	0.01	0.36	0.32
V*_micro_* (cm^3^/g)	-	0.21	0.19
V*_meso_* (cm^3^/g)	-	0.15	0.13
Yield (%)	-	38.6	41.5
dp (nm)	-	1.54	1.42

## Data Availability

Not applicable.
